# 

**Published:** 1895-10-12

**Authors:** 


### CONTENTS.

**The Examinations of the Future** - 21

**The Fallacies of Crude Statistics** - 22

**The Study of Man in the British Association** - 23

**Annotations.**—The Dangers of Street Cycling—Relieving Officers and Medical Extras—Penny Bottles of Sterilised Milk - 25

**Medical Progress and Hospital Clinics—**

The Treatment of Patients after Abdominal Operations - 27

Progress in Medicine.—Diseases of the Stomach (*continued*) - 28

Progress in Surgery.—Surgery of the Liver and Gall Bladder - 29

Progress in Laryngology - 31

New Appliances and Things Medical - 32

**Early Marriages: Royal and Otherwise** - 24

**Notes and News** - 26

**The Sir Andrew Clark Memorial** - 32

**The Institutional Workshop—**

Hospital Expenditure—The Commissariat. II.—The Cost of Provisions per Bed - 33

Practical Departments.—Method of Disinfection - 34

The Story of the Insane from Year to Year - 35

Construction Notes - 36

**Around the Hospitals** - 36

**Scraps and Gleanings** - 37

### EXTRA SUPPLEMENT.—THE NURSING MIRROR.

**News from the Nursing World.**—Sir Edwin Arnold on Nurses—Khama at the London Hospital—Help Wanted for Plaistow—Clean Toys and Light Books—Guy's Hospital Training Schools—Women Guardians in the North—Liverpool Children's Hospital—Nurses for the Portsea Paupers—Irish Fever Nurses—Training at Glasgow Royal Infirmary—Brechin's Charity Demonstration—Across the Channel—District Nurses in Devon—Nursing in Homes—Short Items - xiii
**Minor Appointments** - xv
**Bedford College for Women, London** - xvi
**Elementary Physiology for Nurses.** By C. F. Marshall, late Surgical Registrar, Hospital for Sick Children, Great Ormond Street. X.—The Digestive System - xv
**Florence Nightingale.** By Sir Edwin Arnold - xvi
**"The Hospital" Convalescent Fund** - xvi
**Notes and Queries** - xvi
**Royal British Nurses' Association** - xvi
**A Visit to the Pasteur Institute** - xvii
**Everybody's Opinion** - xvii
**Appointments** - xviii

